# Supplementary material for: Integrative Transcriptomic and Metabolomic Analysis Provides New Insights into the Multifunctional ARGONAUTE 1 Through an Arabidopsis ago1-38 Mutant with Pleiotropic Growth Defects
Source: Plants (Basel). 2025 Dec 23;15(1):44. doi: 10.3390/plants15010044 (PMC12787336; doi:10.3390/plants15010044)
Supplement: Supplementary file 1 [file plants-15-00044-s001.zip › Supplementary files/Supplementary figures.docx]

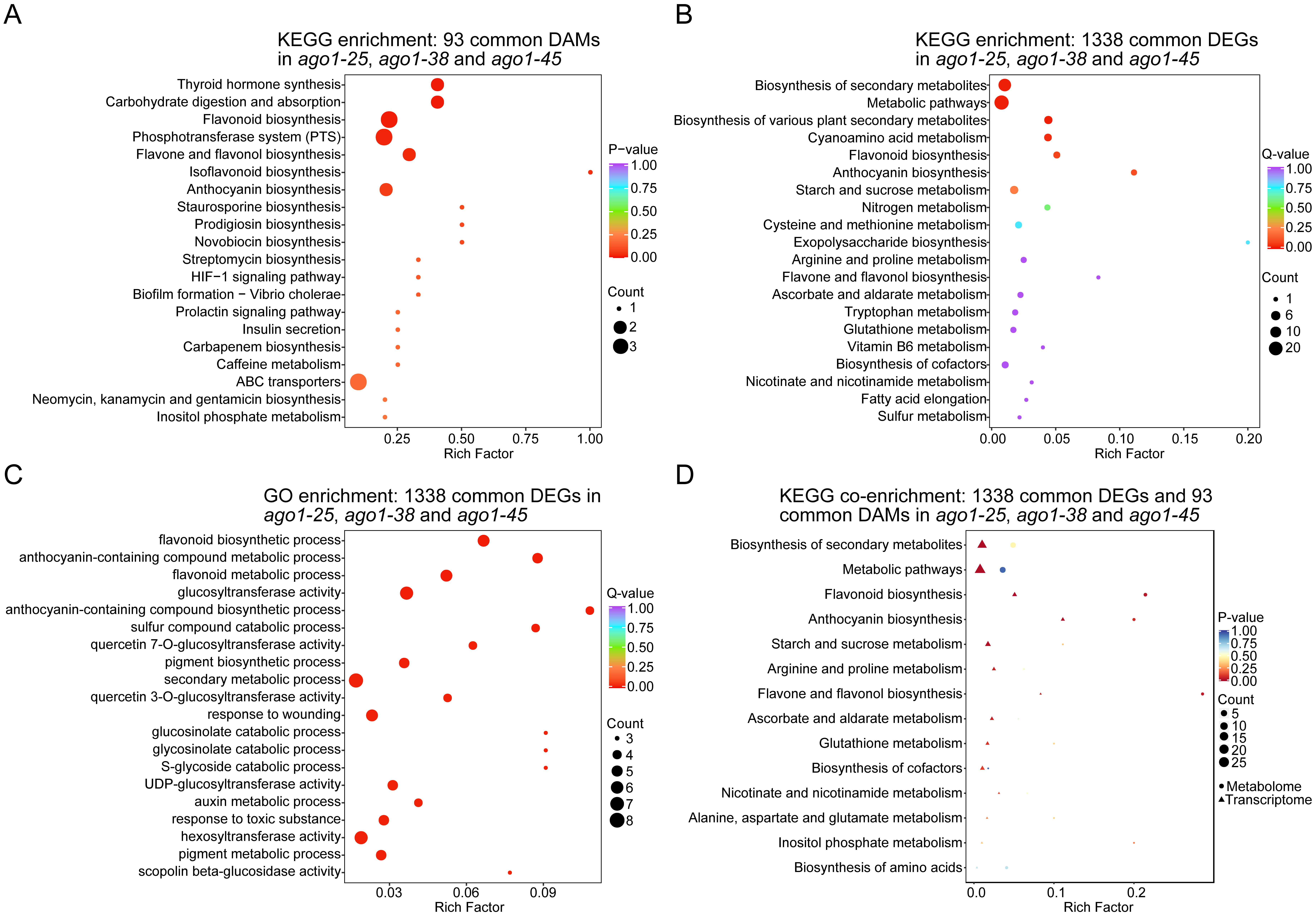


**Figure S1. Analysis of common DAMs and DEGs in three *ago1* mutants.** **A.** KEGG analysis of 93 common DAMs in three *ago1* alleles. **B.** KEGG analysis of 1338 common DEGs in three *ago1* alleles. **C.** GO analysis of 1338 common DEGs in three *ago1* alleles. **D.** KEGG co-enrichment analysis of 1338 common DEGs and 93 common DAMs in three *ago1* alleles.


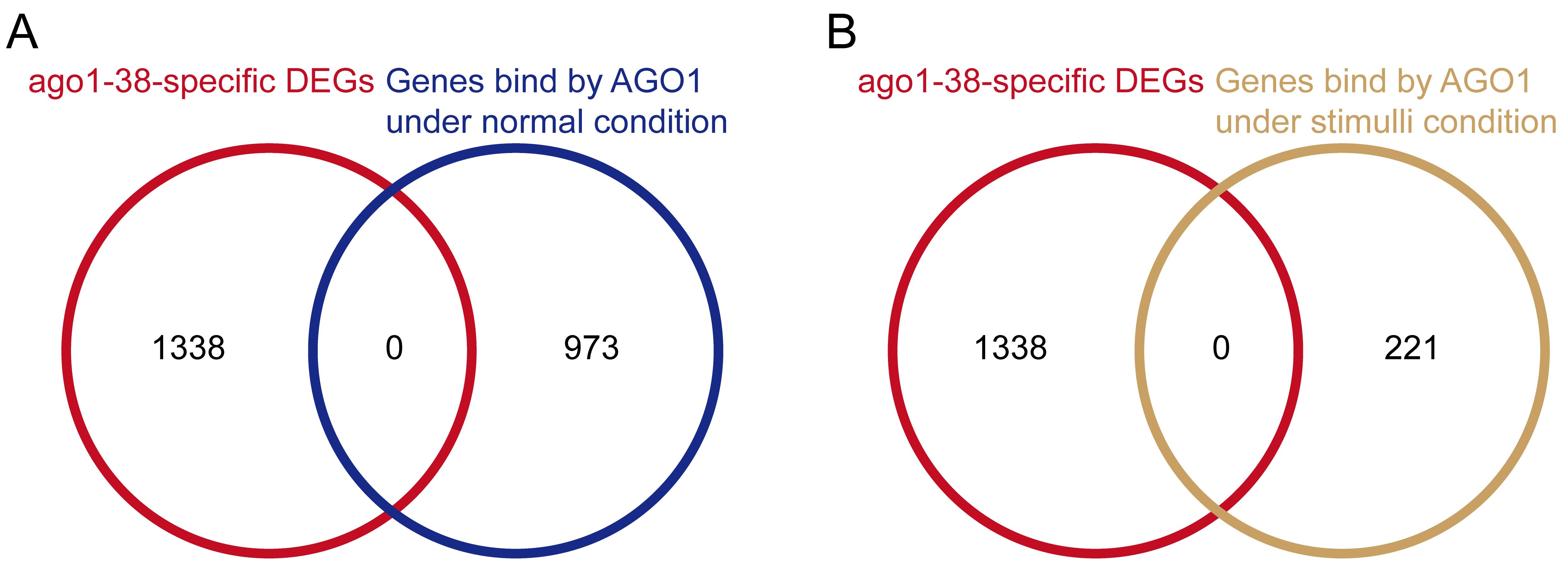


**Figure S2. G186R substitution in *ago1-38* doesn’t affect its gene-binding and transcriptional activation function. A.** Venn diagram comparing *ago1-38*-specific DEGs with previously identified AGO1-binding genes under normal conditions. **B.** Venn diagram comparing *ago1-38*-specific DEGs with previously identified AGO1-binding genes under stimuli conditions.
